# Tick-borne Encephalitis Virus in Wild Rodents in Winter, Finland, 2008–2009

**DOI:** 10.3201/eid1701.100051

**Published:** 2011-01

**Authors:** Elina Tonteri, Anu E. Jääskeläinen, Tapani Tikkakoski, Liina Voutilainen, Jukka Niemimaa, Heikki Henttonen, Antti Vaheri, Olli Vapalahti

**Affiliations:** Author affiliations: University of Helsinki, Helsinki, Finland (E. Tonteri, A.E. Jääskeläinen, L. Voutilainen, A. Vaheri, O. Vapalahti);; Karolinska Institutet, Solna, Sweden (E. Tonteri);; Keski-Pohjanmaa Central Hospital, Kokkola, Finland (T. Tikkakoski);; Finnish Forest Research Institute, Vantaa, Finland (L. Voutilainen, J. Niemimaa, H. Henttonen);; Hospital District of Helsinki and Uusimaa, Helsinki (O. Vapalahti)

**Keywords:** Tick-borne encephalitis virus, rodent, persistence, Finland, TBEV-Eur, TBEV-Sib, Myodes glareolus, Microtus agrestis, viruses, dispatch

## Abstract

Rodents might maintain tick-borne encephalitis virus (TBEV) in nature through latent persistent infections. During 2 subsequent winters, 2008 and 2009, in Finland, we detected RNA of European and Siberian subtypes of TBEV in *Microtus agrestis* and *Myodes glareolus* voles, respectively. Persistence in rodent reservoirs may contribute to virus overwintering.

Tick-borne encephalitis (TBE) is a zoonotic disease endemic to a wide zone, from central and northern Europe to Siberia and Japan (*1*). The causative agent, tick-borne encephalitis virus (TBEV), is maintained in a cycle including ticks and their vertebrate hosts. Ticks serve as vectors and remain infected throughout their life cycle (transstadial transmission). Ticks may acquire the virus when they ingest blood from a viremic host. However, transmission of the virus from infected to uninfected ticks also occurs in the skin of vertebrate hosts, through migratory cells. This process, known as cofeeding, is considered to contribute to the natural cycle of the virus (*2*). Transovarial transmission of TBEV in ticks has also been reported (*3*). In addition to serving as hosts for cofeeding ticks, rodents have been considered to play a role in maintaining TBEV in nature through latent persistent infections, at least for the Siberian subtype (*4*), although strain and subtype differences may exist.

The 3 known subtypes of TBEV are European (TBEV-Eur), Siberian (TBEV-Sib), and Far-Eastern (TBEV-FE) (*5*). TBEV-Sib and TBEV-FE, carried by *Ixodes persulcatus* ticks, are monophyletic; TBEV-Eur and louping ill virus, spread mainly by *I. ricinus* ticks*,* are closely related to each other (*6*). Phylogenetic analyses suggest that TBEV-Sib and TBEV-FE subtypes evolved thousands of years ago, whereas TBEV-Eur has diversified more recently, ≈300 years ago (N.Y. Uzcátegui et al., unpub. data).

TBEV-Eur has a focal distribution, which is dependent on local climatic conditions (*7*). TBEV-Sib and TBEV-FE seem to be spread more widely throughout the *I. persulcatus* tick range (*1*). We studied the persistence of TBEV in wild rodents in natural TBE foci.

## The Study

**Figure 1:**
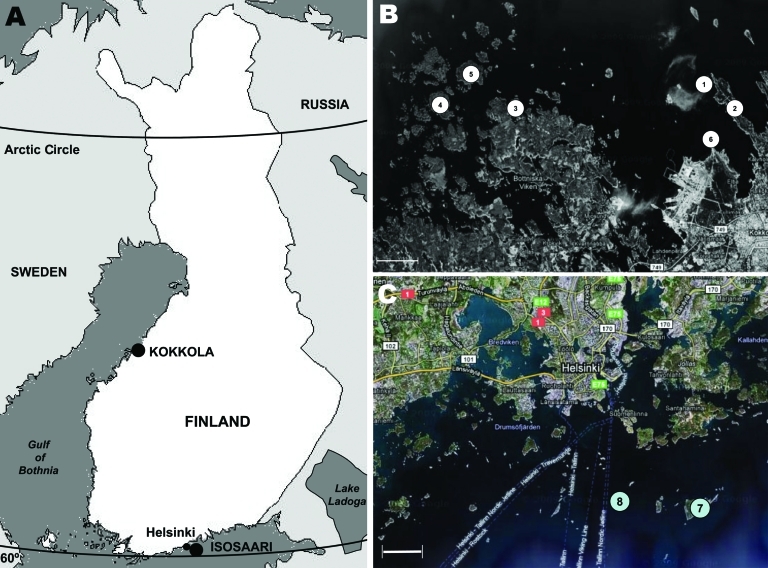
Sites at which rodents were trapped during winters of 2008 and 2009, Finland. A) Locations of trapping sites within Finland. B) Kokkola archipelago, where Siberian subtype of tick-borne encephalitis virus is endemic: 1,Trullevi, Kupu Island; 2,Trullevi; 3, Enträskholmen Island; 4, Börskär Island; 5, Norra Hamnskäret Island; 6, Harrbådan. C) Helsinki archipelago, Isosaari, where European subtype of tick-borne encephalitis virus is endemic: 7, Isosaari Island; 8, Harmaja Island. Scale bars indicate 2 km.

**Figure 2:**
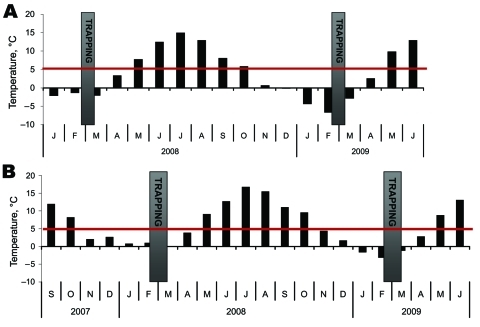
Monthly day and night mean temperatures at the trapping sites. Daily maximum temperatures had not reached 5°C for >50 days before trapping. Tick-feeding season is considered to begin when temperature in the ground reaches the tick activity limit and stays above it (*1*). A) Kokkola archipelago, where Siberian subtype of tick-borne encephalitis virus is endemic. B) Helsinki archipelago, where European subtype of tick-borne encephalitis virus is endemic. Although trapping was conducted on Isosaari, temperature data were unavailable and were instead collected on Harmaja, a nearby island (Figure 1). Gray bars indicate time of trapping; red line indicates tick activity limit. Data source: Finnish Meteorological Institute (http://ilmatieteenlaitos.fi/en/).

Small mammals were collected from 2 sites in Finland, representing TBEV-Eur– (Isosaari Island, Helsinki archipelago) and TBEV-Sib– (Kokkola archipelago) endemic areas (Figure 1). Clinical TBE cases and TBEV-positive ticks have been detected at each of these sites (*8**,**9*). The animals were trapped from February 19 through March 12 in 2008 and 2009, when daily maximum temperatures had been below the tick activity limit for months (Figure 2). Thus, TBEV RNA in rodent tissues would likely have persisted from the previous summer.

Animals were caught in snap traps set overnight and stored at –80°C until they were dissected. Brain, lungs, liver, and spleen were stored at –70°C. Pieces of lungs, liver, and spleen were also pooled for reverse transcription–PCR (RT-PCR). Blood from dissected heart was extracted and stored in phosphate-buffered saline for serologic analysis. A total of 50–100 mg of each tissue sample or organ pool was homogenized in 1 mL of TriPure Isolation Reagent (Roche Diagnostics Corp., Indianapolis, IN, USA) with glass beads and sand in a MagNA Lyser (Roche Diagnostics GmbH, Mannheim, Germany). RNA was extracted by using TriPure Isolation Reagent according to the manufacturer’s instructions.

The RNA was dissolved in 25 µL of diethylpyrocarbonate-treated water and analyzed by using real-time RT-PCR as described by Schwaiger and Cassinotti (*10*), except we used 50 nmol/L forward primer, 300 nmol/L reverse primer, and 200 nmol/L probe. Samples positive by real-time RT-PCR were further analyzed by nested RT-PCR, amplifying a 252-nt sequence from the TBEV nonstructural protein (NS) 5 gene. Analysis was performed as described by Puchhammer-Stöckl et al. (*11*), using modified primer as described by Jääskeläinen et al. (*9*). Dissection of animals and RNA extraction were done in a laboratory in which no TBEV RNA or cDNA had been previously introduced. Diluted blood samples (≈1:10) were studied for antibodies against TBEV by using an immunofluorescence assay with TBEV-Eur–infected Vero E6 cells as antigen and polyclonal rabbit anti-mouse fluorescein isothiocyanate conjugate (Dako, Glostrup, Denmark). Sample and conjugate were incubated for 30 min at 37°C.

During the 2 subsequent years, 202 rodents and insectivores were trapped outside the tick-feeding season (Table). All rodents in the TBEV-Sib focus were bank voles (*Myodes glareolus*), and those in the TBEV-Eur focus were field voles (*Microtus agrestis*). Altogether 23 voles and 1 common shrew *(Sorex araneus)* were positive for TBEV RNA by real-time RT-PCR. Viral RNA was detected mostly in brain (or in brain and internal organs); for 2 voles, it was detected only in internal organs. Of the real-time RT-PCR-positive samples, 5 were also positive with the less sensitive (*10**,**11*) NS5 gene–targeting nested RT-PCR. The 165-nt stretch of the NS5 gene obtained from a bank vole (GenBank accession no. GU458800) from the TBEV-Sib–endemic area (Figure 1) differed 0–4 nt from published sequences from *I. persulcatus* ticks collected from the same area in 2004 (*9*). No sequence from the TBEV-Eur area could be recovered.

**Table:** Small mammals trapped during 2 subsequent winters (2008 and 2009) in TBEV-Eur– and TBEV-Sib–endemic areas, Finland*

Location (virus subtype), year, and mammal species	No. animals trapped	No. RNA positive by real-time PCR			No. (%) antibody positive†
		Brain	Organ pool/spleen‡	Total (**%**)	
Kokkola (TBEV-Sib)					
2008					
*Myodes glareolus* vole	63	1§	0	1 (1.6)	2 (3.2)
*Sorex caecutiens* shrew	4	0	0	0	0
*S. araneus* shrew	7	0	0	0	0
2009					
*M. glareolus* vole	17	4§	2	5 (29.4)	4 (23.5)
*S. araneus* shrew	3	0	1	1 (33.3)	0
Isosaari (TBEV-Eur)					
2008					
*Microtus agrestis* vole	71	3¶	2	4 (5.6)	0
*S. araneus* shrew	7	0	0	0	0
2009					
*M. agrestis* vole	24	13	0	13 (54.2)	2 (8.3)
*S. araneus* shrew	6	0	0	0	0

*TBEV, tick-borne encephalitis virus; TBEV-Eur, European subtype of TBEV; TVEV-Sib, Siberian subtype of TBEV. †Blood samples, diluted ≈1:10 in phosphate-buffered saline, were screened by immunofluorescence assay with TBEV-infected Vero E6 cells as antigen. ‡For animals collected in 2008, organ pool of lungs, spleen, and liver were screened; for animals collected in 2009, only spleen was screened. §Three brain samples positive for TBEV RNA by real-time reverse transcription–PCR (RT-PCR) (1 in 2008 and 2 in 2009) were also positive for the TBEV nonstructural protein (NS) 5 gene by nested RT-PCR. ¶Two brain samples positive for TBEV RNA by RT-PCR were also positive for the TBEV NS5 gene by nested RT-PCR.

Serologic analysis showed that in the TBEV-Eur area, only 2 of 16 mammals whose brain tissue was positive for TBEV RNA had anti-TBEV antibodies; whereas, in the TBEV-Sib area, all 5 rodents whose brain tissue was positive for TBEV RNA had antibodies as well. The difference was significant (Fisher exact test, p = 0.001). One antibody-positive rodent did not have detectable levels of TBEV RNA in brain, lung, liver, or spleen.

## Conclusions

The focal distribution of TBEV-Eur has generally been explained by climatic factors, which define the temporal occurrence of nymphs and larvae and, consequently, the frequency of cofeeding (*12*). TBEV-Sib is also transmitted vertically between generations of adapted reservoir rodents and nonadapted laboratory mice (*13*). Furthermore, virus persistence and latent infections in rodents outside the tick-feeding season may occur in the TBEV-Sib–endemic zone in Siberia. Thus, TBEV-Sib may be less dependent than TBEV-Eur on tick cofeeding, and thereby on the climate, and seems to occur less focally (*4**,**14*). However, TBEV has also been reported to persist over winter in western Slovakia, a TBEV-Eur–endemic area (*15*).

We detected TBEV RNA in brain and internal organ samples of rodents in TBEV-Eur– and TBEV-Sib–endemic areas (Figure 2) several months after tick-feeding season. Almost all TBEV RNA–positive rodents in the TBEV-Sib–endemic area had anti-TBEV antibodies, whereas in the TBEV-Eur area, most did not. This finding might indicate a difference in the infection process and host adaptation between the 2 subtypes. Persistent TBEV has been isolated from rodents in a TBEV-Eur–endemic area even when no antibodies for TBEV were detected (*15*).

The host species at the 2 trapping sites differed. To find bank voles as TBEV-Sib hosts was not unexpected, considering that the congeneric red vole (*Myodes rutilus*) is a common host for TBEV in Siberia (*14*). However, earlier studies have implicated yellow-necked mice (*Apodemus flavicollis*) and bank voles as major hosts for TBEV-Eur (*2*). In our TBEV-Eur focus, all TBEV-positive animals were field voles, which dominated Isosaari Island in the absence of *Myodes* and *Apodemus* spp. rodents.

Our results show that TBEV-Sib and TBEV-Eur may cause prolonged latent infections in host rodents. We detected TBEV RNA in brain and other tissues from rodents in some of the northernmost TBEV-endemic areas in Europe, where the daily maximum temperatures and the snow cover in winter do not enable nymphal or larval activity. Further comparative studies are needed to explain the type of latency and its possible role in the ecology of different subtypes of TBEV.
